# Associating bovine herpesvirus 1 envelope glycoprotein gD with activated phospho-PLC-γ1(S1248)

**DOI:** 10.1128/spectrum.01963-23

**Published:** 2023-09-01

**Authors:** Chang Liu, Weifeng Yuan, Hao Yang, Junqing Ni, Linke Tang, Heci Zhao, Donna Neumann, Xiuyan Ding, Liqian Zhu

**Affiliations:** 1 College of Life Sciences, Hebei University, Baoding, China; 2 Institute of Animal Sciences, Chinese Academy of Agricultural Sciences, Beijing, China; 3 Animal Husbandry and Improved Breeds Work Station of Hebei Province, Shijiazhuang, China; 4 Department of Ophthalmology and Visual Sciences, University of Wisconsin-Madison, Madison, Wisconsin, USA; 5 Key Laboratory of Microbial Diversity Research and Application of Hebei Province, College of Life Science, Hebei University, Baoding, China; Oklahoma State University College of Veterinary Medicine, Stillwater, Oklahoma, USA

**Keywords:** bovine herpesvirus 1, phospholipase C-γ1, Golgi apparatus, gD

## Abstract

Phospholipase C gamma 1 (PLC-γ1) may locate at distinct subcellular locations, such as cytosol, plasma membrane, and nucleus for varied biological functions. Bovine herpesvirus 1 (BoHV-1) productive infection activates PLC-γ1 signaling, as demonstrated by increased protein levels of phosphorylated-PLC-γ1 at Ser1248 [p-PLC-γ1(S1248)], which benefits virus productive infection. Here, for the first time, we reported that Golgi apparatus also contains activated p-PLC-γ1(S1248). And BoHV-1 productive infection at later stages (24 hpi) increased the accumulation of p-PLC-γ1(S1248) in the Golgi apparatus, where p-PLC-γ1(S1248) forms highlighted puncta observed via a confocal microscope. Coimmunoprecipitation studies demonstrated that the Golgi p-PLC-γ1(S1248) is specifically associated with the viral protein gD but not gC. In addition, we found that p-PLC-γ1(S1248) is consistently associated with both the plasma membrane-associated virions and the released virions. When the virus-infected cells were treated with PLC-γ1-specific inhibitor, U73122, for a short duration of 4 hours prior to the endpoint of virus infection, we found that the viral protein gD was trapped in the Golgi apparatus, suggesting that the PLC-γ1 signaling may facilitate trafficking of progeny virions out of this organelle. These findings provide a novel insight into the interplay between PLC-γ1 signaling and BoHV-1 replication.

**IMPORTANCE:**

Bovine herpesvirus 1 (BoHV-1) productive infection increases protein levels of phosphorylated-phospholipase C gamma 1 at Ser1248 [p-PLC-γ1(S1248)]. However, whether it causes any variations to p-PLC-γ1(S1248) localization is not well understood. Here, for the first time, we found that partial p-PLC-γ1(S1248) is residing in the Golgi apparatus, where the accumulation is enhanced by virus infection. p-PLC-γ1(S1248) is consistently associated with virions, partially via binding to gD, in both the Golgi apparatus and cytoplasm membranes. Surprisingly, it also associates with the released virions. Of note, this is the first evidenced BoHV-1 virion-bound host protein. It seems that p-PLC-γ1(S1248) works as an escort during trafficking of progeny virions out of Golgi apparatus to the plasma membranes as well as releasing outside of the cell membranes. Furthermore, we showed that the activated p-PLC-γ1(S1248) is potentially implicated in the transport of virions out of Golgi apparatus, which may represent a novel mechanism to regulate virus productive infection.

## INTRODUCTION

Bovine herpesvirus 1 (BoHV-1) belongs to the family *Herpesviridae* and the subfamily *Alphaherpesvirinae*, which is one of the most important pathogens of cattle that causes inflammatory diseases in multiple systems (1, 2). The acute virus infection may lead to inflammatory lesions in the upper respiratory tract, which may result in subsequent invasion by other pathogens (3 – 5). Thereby, bovine respiratory disease complex, a life-threatening disease of cattle, tends to be developed (6). Apart from respiratory disease, it is also closely associated with abortion (6, 7). It has been documented that BoHV-1 infection-associated diseases cost the US cattle industry ~$3 billion annually (8).

Phospholipase C gamma 1 (PLC-γ1), a ubiquitous molecule, belongs to the PLC family, which consists of 6 classes (β, γ, δ, ε, η, and ζ) and a total of 13 members in humans. PLC-γ1 serves as a downstream signal molecule of receptor tyrosine kinase that regulates various cellular signaling pathways (9). For example, PLC-γ1 forms a complex with epidermal growth factor (EGF) receptors, and undergoes phosphorylation of PLC-γ1 at Tyr771, Tyr783, and Ser1248 in response to EGF stimulation, which activates PLC-γ1 enzymatic activity (10). In mechanism, the phosphorylation of PLC-γ1 at Y783 by EGF receptors (EGFR) causes a conformational change of PLCγ1 allowing the interaction with Akt, and phosphorylation of PLC-γ1 at S1248 by Akt (11), and the activated PLC-γ1(S1248) further stimulates Akt signaling transduction (12).

We have previously reported that PLC-γ1(S1248) is potentially involved in cell adhesion (13), and the phosphorylation of PLC-γ1 at S1248 is important for BoHV-1 productive infection, which, in turn, benefits virus infection (14, 15). And the PLC-γ1(S1248) is potentially involved in BoHV-1 infection-induced inflammatory responses via promoting the production of inflammation-related mediators, reactive oxidative species, and activation of inflammation-related MAPK signaling pathways (14). In addition, the phosphorylation of PLC-γ1 at S1248 is stimulated by proinflammatory cytokines, such as tumor necrosis factor alpha (TNF-α) and interleukin-1β (IL-1β) (16); thereby, a positive feedback loop between PLC-γ1 signaling and pro-inflammatory cytokines is potentially established, which may boost the pathogenesis of the inflammatory disease (17). So, PLC-γ1(S1248) is potentially implicated in the pathogenicity of various inflammatory diseases. Currently, how the activated p-PLC-γ1(S1248) is mobilized following virus productive infection in cell culture remains to be determined.

It has been reported that the activated PLC-γ1 hydrolyzes phosphatidylinositol 4,5-bisphosphate to generate diacylglycerol (DAG) and inositol triphosphate (IP3) (18, 19). Concomitantly, p-PLC-γ1(S1248) translocates from the cytosol to the particulate cell fraction via association with PLC-γ1-specific anchoring proteins (20). It has been established that protein kinase D (PKD) is recruited into the *trans*-Golgi network (TGN) by DAG, where it plays a central role in the transport of cellular cargos from the TGN to the cell surface (21). For example, the trafficking of herpes simplex virus type 1 (HSV-1) capsids from the TGN to plasma membranes is largely dependent on PKD, which is also affected by DAG (22). In view of the fact that DAG, one of the PLC-γ1-catalyzed products, has critical effects on intracellular cargo trafficking, and BoHV-1 productive infection at later stages leads to sustained activation of PLC-γ1 (14, 15). So we hypothesized that PLC-γ1 signaling may benefit intracellular trafficking of BoHV-1 virions.

In this study, subcellular localization of the activated PLCγ1 proteins, p-PLCγ1(S1248), stimulated by BoHV-1 infection at the later stages was extensively characterized. For the first time, we found that the activated p-PLCγ1(S1248) stimulated by the virus infection mainly resides in the Golgi apparatus, where it interacts with virion-associated proteins, and assembles clusters of highlighted puncta characterized by confocal microscope. In addition to the Golgi virions, partial of p-PLCγ1(S1248) is associated with both cellular membrane-associated virions and the released virions. Importantly, we found that the treatment of virus-infected cells by using PLC-γ1-specific inhibitor U73122 trapped gD in the Golgi apparatus, suggesting that PLC-γ1 signaling may facilitate virus trafficking out of Golgi apparatus.

## RESULTS

### BoHV-1 productive infection induces formation of p-PLC-γ1(S1248) puncta

**Fig 1:**
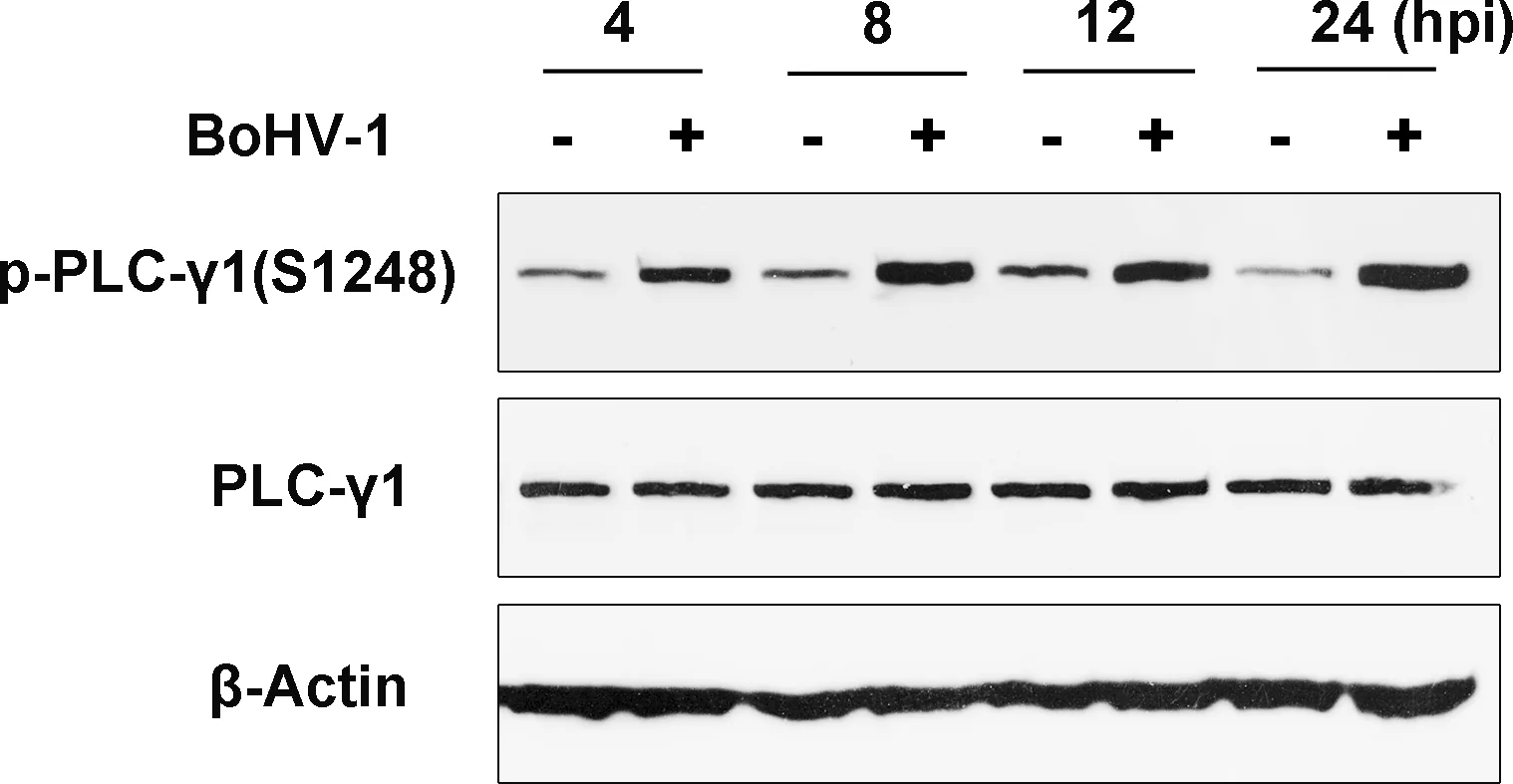
BoHV-1 infection in MDBK cells enhanced the phosphorylation of PLC-γ1 at S1248. MDBK cells were infected with BoHV-1 at an MOI of 0.1, and at indicated time points, the cell lysates were prepared for Western blotting. The data shown are representative results of two independent experiments.

**Fig 2:**
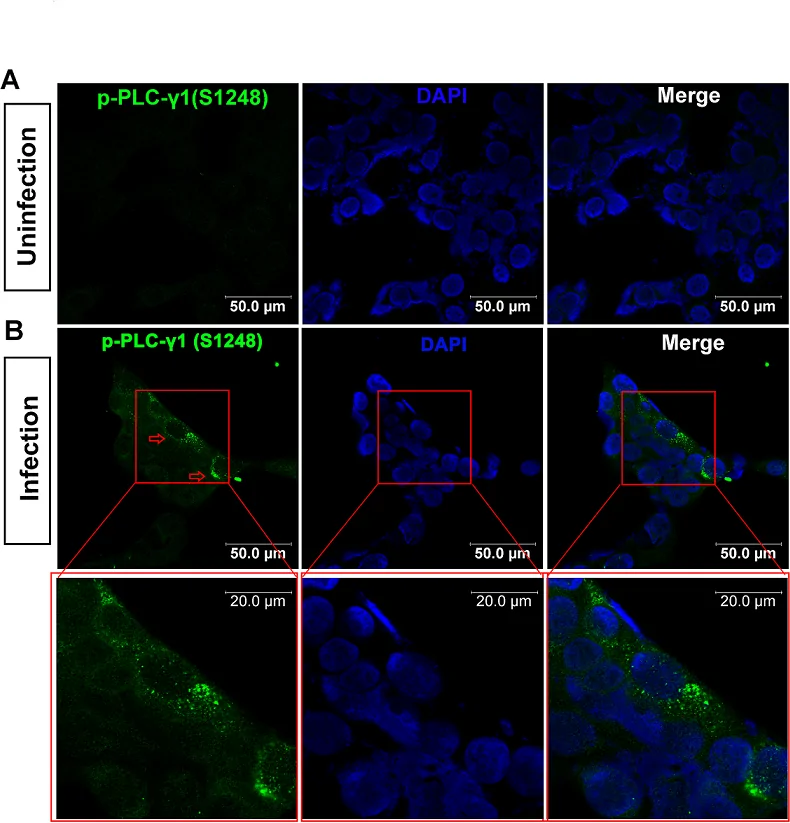
Subcellular localization of p-PLC-γ1(S1248) in BoHV-1-infected MDBK cells. MDBK cells were either mock infected (A) or infected (B) with BoHV-1 (MOI = 0.1) for 24 hours. Then they were stained with an antibody against p-PLC-γ1(S1248) (green). Nuclei were stained with DAPI (4′,6-diamidino-2-phenylindole) (blue). Then the immunostaining was visualized, and images were captured using confocal microscopy. Arrow indicates p-PLC-γ1(S1248) puncta. Zoom-in images in red frames demonstrated the typical puncta. These images are representative of those from three independent experiments.

BoHV-1 productive infection in Madin-Darby bovine kidney (MDBK) cells leads to sustained activation of PLC-γ1, as demonstrated by increased protein levels of phosphorylated-PLC-γ1 at Ser1248 [p-PLC-γ1(S1248)] (Fig. 1), which is in line with our previous report (14). We are interested in how PLC-γ1 is mobilized following virus infection at 24-hour post-infection (hpi). We chose the later stages of virus infection of 24 hpi because the progeny virions are usually produced and released in large amounts (data not shown), and the protein levels of p-PLC-γ1(S1248) peaked at this time point (Fig. 1), which are required to extensively reflect the interaction between p-PLC-γ1(S1248) and virus infection. We initially performed immunofluorescence assays (IFAs) to explore the subcellular localization of p-PLC-γ1(S1248). As expected, immunostaining of p-PLC-γ1(S1248) was readily detected in the virus-infected cells but not in the mock-infected control (Fig. 2), which corroborated our findings of Western blot as shown in Fig. 1. Of note, highlighted p-PLC-γ1(S1248) puncta were readily visualized in virus-infected MDBK cells (Fig. 2B, denoted by arrows). Taking these data together, BoHV-1 productive infection at later stages not only increased the protein levels of p-PLC-γ1(S1248) but also induced the formation of typical clusters of p-PLC-γ1(S1248) puncta.

### BoHV-1 productive infection promotes accumulation of p-PLC-γ1(S1248) in the Golgi apparatus

**Fig 3:**
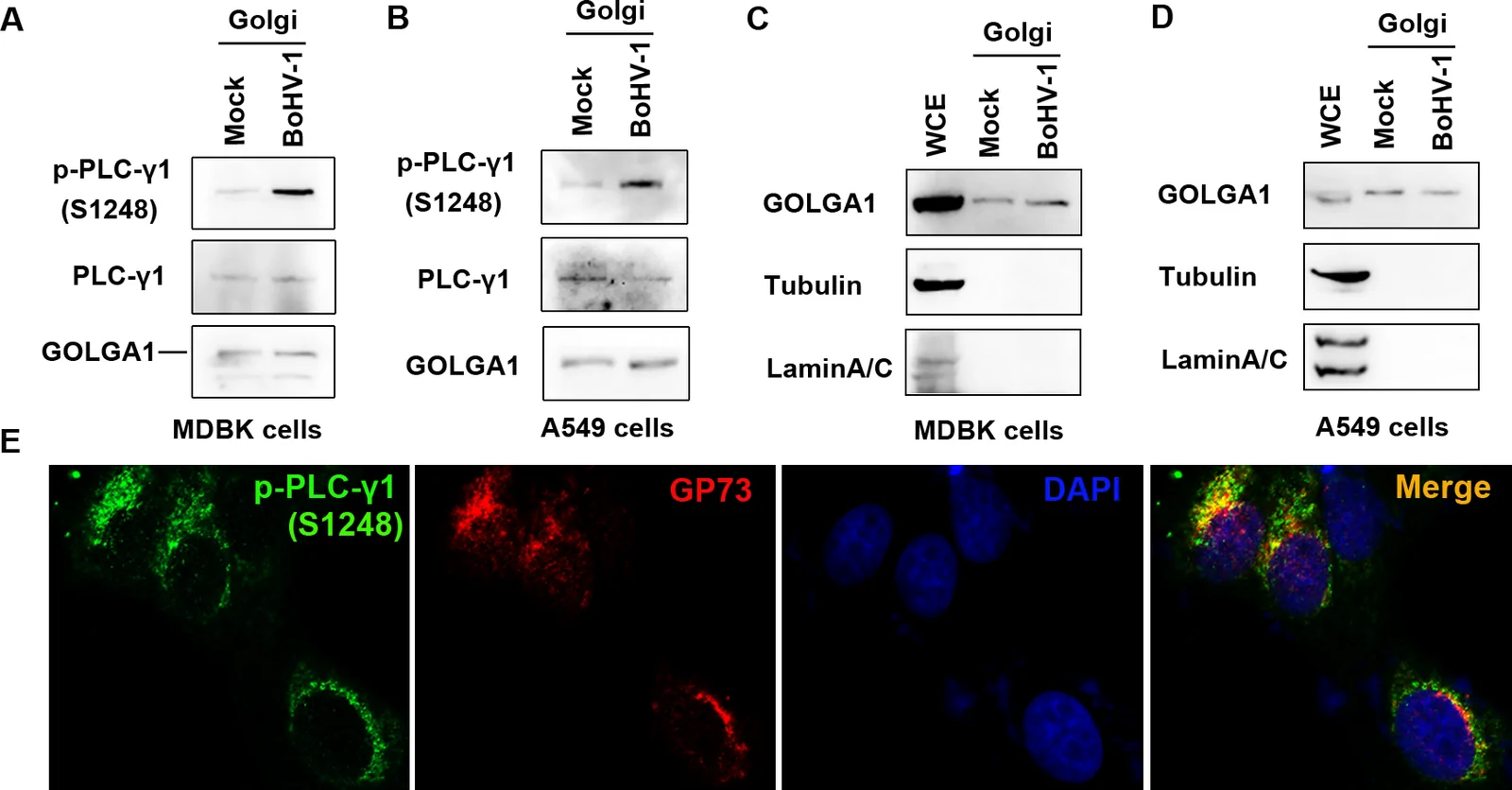
BoHV-1 infection had effects on the accumulation of p-PLC-γ1(S1248) in the Golgi apparatus. MDBK cells (A and C) and A549 cells (B and D) in 60 mm dishes were mock infected or infected with BoHV-1 (MOI = 0.1) for 24 hpi. Then the Golgi apparatus was purified using a commercial kit (Beijing Bio Lebo Technology, cat# HR0247), and the protein levels of p-PLC-γ1(S1248), PLC-γ1, LaminA/C, tubulin, and GOLGA1 were detected by immunoblots. The protein levels of GOLGA1 were probed as the protein loading control. (E) After infection for 24hours, BoHV-1-infected MDBK cells (MOI = 0.1) were stained with antibodies against p-PLC-γ1(S1248) (green) and Golgi-specific marker GP73 (red). Nuclei were stained with DAPI (blue). Then the immunostaining was visualized, and images were captured using confocal microscopy. The data shown are representative of three independent experiments.

The profile of p-PLC-γ1(S1248) puncta induced by virus infection is reminiscent of the Golgi apparatus as reported elsewhere (23). To determine whether partial p-PLC-γ1(S1248) was stimulated by BoHV-1 infection localized at Golgi apparatus, cellular fractions of Golgi apparatus in MDBK cells were isolated by using a commercial Golgi apparatus purification kit, and then Western blotting was performed to detect the protein of p-PLC-γ1(S1248). The Golgin A1 (GOLGA1) protein, a marker of Golgi apparatus, was employed as a protein loading control. As a result, both PLC-γ1 and p-PLC-γ1(S1248) were readily detected in the Golgi fractions of either mock-infected or virus-infected MDBK cells (Fig. 3A). And more protein levels of p-PLC-γ1(S1248) were detected, in comparison to the mock-infected controls. Since the steady-state protein levels of total PLC-γ1 in Golgi fractions were not obviously increased following virus infection (Fig. 3A), we suggested that the virus productive infection at later stages enhanced PLC-γ1 activity in Golgi apparatus via increased phosphorylation.

In addition to MDBK cells, human lung carcinoma cell line A549 also supports BoHV-1 productive infection, and virus infection also stimulates PLC-γ1 signaling for efficient replication (24). As observed in MDBK cells, both PLC-γ1 and p-PLC-γ1(S1248) were readily detected in the isolated Golgi apparatus of A549 cells, and virus infection for 24 hours increased the protein levels of p-PLC-γ1(S1248) but decreased that of total PLC-γ1 (Fig. 3B), suggesting that virus productive infection in A549 cells also increased the activity of PLC-γ1. A further study showed that neither Tubulin nor LaminA/C, a marker for the extractions of cytosol and nucleus, respectively, was readily detected by Western blot (Fig. 3C and D), which confirmed that the Golgi fractions from both MDBK and A549 cells were not contaminated by the cytosol protein and nuclear protein, which validated the findings that the virus infection increased the protein levels of p-PLC-γ1(S1248) in the Golgi apparatus.

Because the commercially available antibodies against p-PLC-γ1(S1248) and GOLGA1 are both produced in rabbits, they are not allowed to visualize both p-PLC-γ1(S1248) puncta and the Golgi apparatus in the same cells via IFA study. Thus, a commercially available mouse monoclonal antibody (mAb) against GP73(GOLPH2), an alternate marker of Golgi membrane protein, was employed for a further IFA, which enable us to detect the Golgi apparatus and p-PLC-γ1(S1248) puncta via double staining. Indeed, the IFA analysis further confirmed that partial p-PLC-γ1(S1248) puncta induced by virus productive infection at later stages (24 hpi) are located at the Golgi apparatus (Fig. 3E).

Taken together, for the first time, we found that a subset of PLC-γ1 molecules is located at the Golgi apparatus, where its activity is dramatically enhanced during BoHV-1 productive infection at later stages without cell type-dependent manners.

### GOLGA1, a specific marker of the Golgi apparatus, colocalizes with BoHV-1 glycoprotein gD

**Fig 4:**
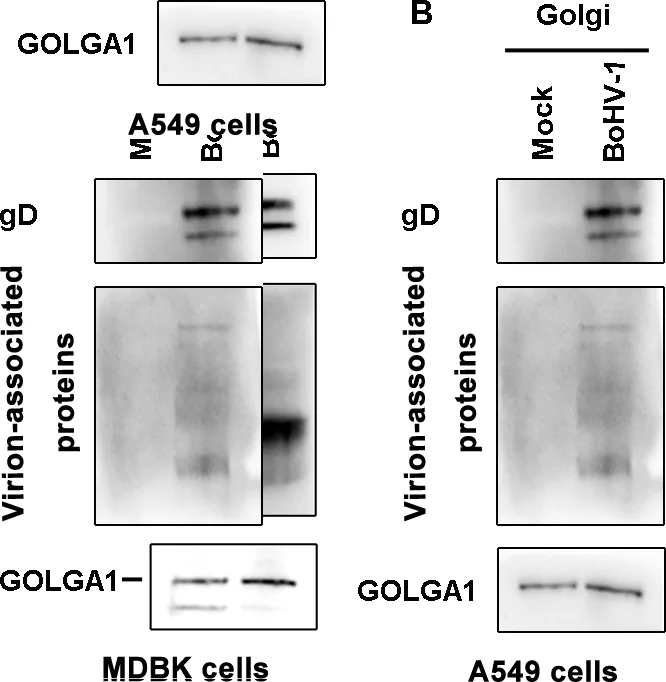
Detection of BoHV-1 virion-associated proteins in Golgi apparatus. MDBK cells (A) and A549 cells (B) in 60 mm dishes were either mock infected or infected with BoHV-1 (MOI = 0.1) for 24 hpi. Then Golgi apparatus was purified using a commercial kit (Beijing Bio Lebo Technology, cat# HR0247). Then the viral envelope glycoprotein gD (upper panels) and virion-associated proteins (middle panels) were detected by immunoblots. The protein levels of GOLGA1 were probed as the protein loading control. The data shown are representative of three independent experiments.

It has been reported that virions of HSV-1 localize to the Golgi apparatus, which enables virus packaging, envelopment, and trafficking to the cytoplasm (25). Like HSV-1, Golgi apparatus is the essential site for BoHV-1 packaging into mature virions (26). Here, Western blotting analysis was performed to detect viral proteins in the isolated Golgi apparatus via using a monoclonal antibody against virus glycoprotein gD and a polyclonal antibody (pAb) against virion-associated proteins, respectively. As a result, both gD and virion-associated proteins were detected from the Golgi fractions originating from virus-infected MDBK cells (Fig. 4A). Similar to the observations in MDBK cells, both gD and virion-associated proteins were readily detected from the Golgi fractions derived from the virus-infected A549 cells (Fig. 4B). These data indicated that the part of BoHV-1 virions also arrived in Golgi apparatus during virus productive infection at later stages without cell type-dependent manners.

**Fig 5:**
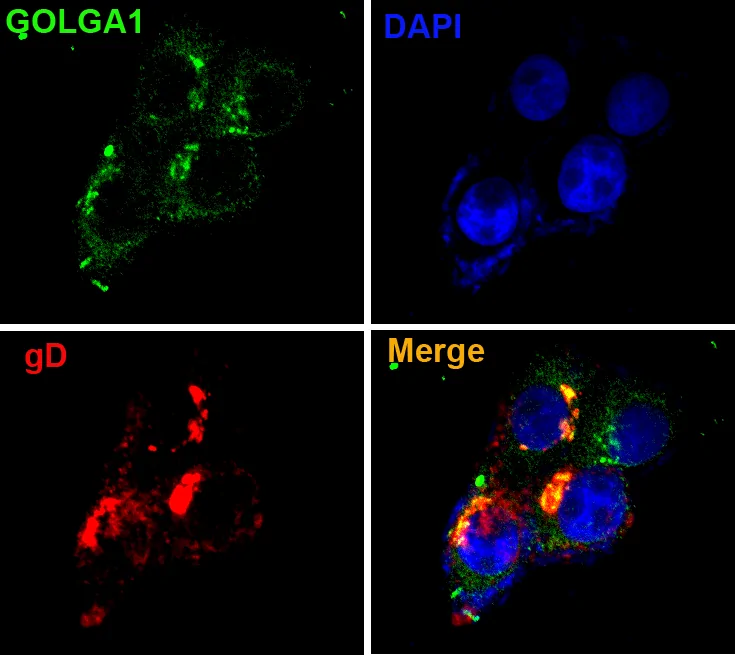
Detection of BoHV-1 viral protein gD and GOLGA1 in virus-infected MDBK cells with IFA. After infection with BoHV-1 (MOI = 0.1) for 24 hours, MDBK cells were immunostained using GOLGA1 antibody (green), a marker protein of the Golgi apparatus, and an antibody against viral protein gD (red) and then visualized by confocal microscopy. Nuclei were stained with DAPI (blue). The images were captured using confocal microscopy. These images are representative of those from three independent experiments.

IFA analysis was performed as an independent method to confirm that the viral protein gD locates in the Golgi apparatus. GOLGA1 was employed as a specific marker of the Golgi apparatus. As a result, GOLGA1 puncta representing Golgi apparatus were readily observed in virus-infected cells (at 24 hpi), and the majority of viral glycoprotein D (gD) colocalize well with GOLGA1 (Fig. 5), confirming that gD molecules may reside in the Golgi apparatus. Taken together, a large amount of virion-associated proteins, such as gD, locate in the Golgi apparatus during virus productive infection at later stages, and the gD can be used as a potential indicator of virus-infected Golgi apparatus.

### p-PLC-γ1(S1248) associates with virion-associated proteins in the Golgi apparatus

**Fig 6:**
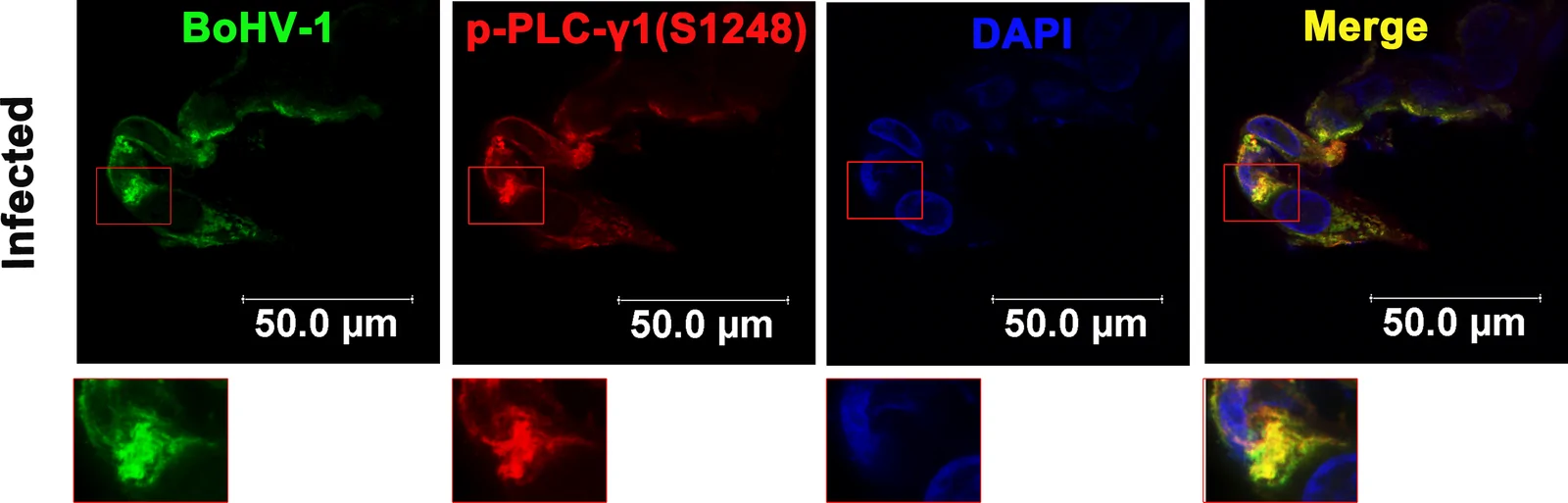
Detection of p-PLC-γ1(S1248) and virion-associated proteins in virus-infected MDBK cells with IFA. After infection with BoHV-1 (MOI = 0.1) for 24 hours, MDBK cells were immunostained via using an antibody against virion-associated proteins (green), and p-PLC-γ1(S1248) monoclonal antibody (red), then visualized by confocal microscopy. Nuclei were stained with DAPI (blue). The images were captured using confocal microscopy. These images are representative of those from three independent experiments.

Since our foregoing data indicate that a subset of p-PLC-γ1(S1248) and virion-associated proteins reside in the Golgi apparatus during BoHV-1 productive infection at the later stages (Fig. 3 and 4), IFA analysis was performed to characterize their subcellular localization. Interestingly, we found that the virion-associated proteins colocalize well with that of the p-PLC-γ1(S1248) puncta (Fig. 6). Of note, our data have indicated that virion-associated proteins, such as gD, can be used as a potential indicator of virus-infected Golgi apparatus, which corroborate our findings that the p-PLC-γ1(S1248) puncta are located at the Golgi apparatus.

**Fig 7:**
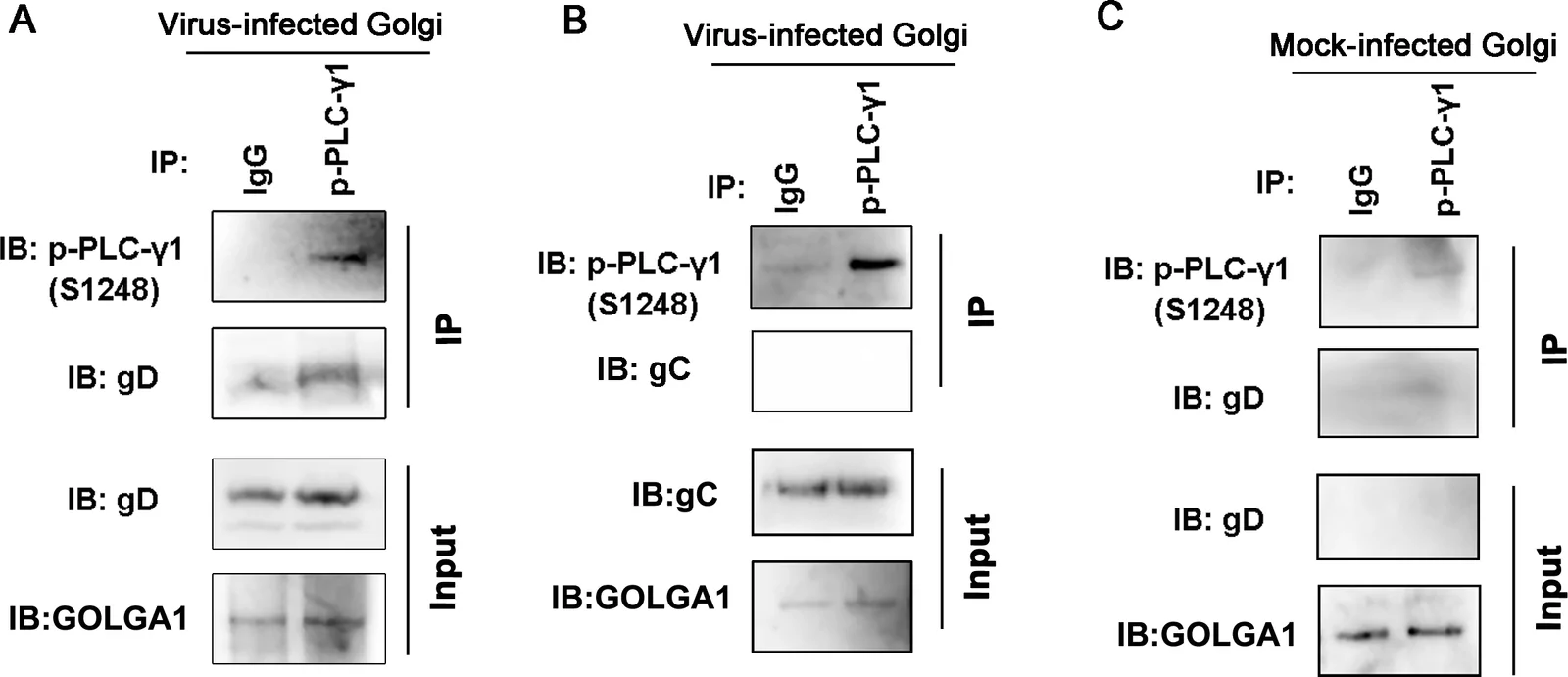
Detection of the association between viral protein gD and p-PLC-γ1(S1248) with IP. MDBK cells were infected with BoHV-1 (MOI = 0.1) for 24 hours. The Golgi apparatus was isolated by using a commercial purification kit (Beijing Biolabo Technology, cat# HR0247). Then the cell lysates were subjected to IP using the antibody against p-PLC-γ1(S1248), and the isotype IgG, which was used as a control. The collected immunoprecipitates were subjected to Western blot to detect indicated proteins of either gD (A) or gC (B). Viral protein of either gD (A) or gC (B) was detected from the input samples, which was used as an indicator of virus infection. The Golgi apparatus isolated from mock-infected cells were subjected to IP using the antibody against p-PLC-γ1(S1248), and the isotype IgG, which was used as a control. Then gD protein levels were detected from both the immunoprecipitates and the input samples by Western blot (C). The protein of GOLGA1, a marker of Gogi apparatus was detected from the input samples, serving as an indicator of protein loading control (A, B and C). These data shown are representative of those from three independent experiments.

To investigate whether p-PLC-γ1(S1248) interacts with the viral proteins in the Golgi apparatus, immunoprecipitation (IP) studies were initially performed with cellular fractions of the virus-infected Golgi apparatus. A p-PLC-γ1(S1248)-specific monoclonal antibody was used to immunoprecipitate proteins associated with p-PLC-γ1(S1248), and the isotype IgG was included as a control. Following separation of the immunoprecipitated proteins in an SDS-polyacrylamide gel, Western blotting assays were performed to identify proteins associated with p-PLC-γ1(S1248). These studies, which were performed using gD monoclonal antibody, revealed that gD is associated with p-PLC-γ1(S1248) (Fig. 7A). Specific binding to the antibody in these IP assays was confirmed by isotype IgG (Fig. 7A), validating that the target proteins can be specifically precipitated by the p-PLC-γ1(S1248) antibody. We also tested whether the viral envelope glycoprotein gC was associated with p-PLC-γ1(S1248). However, gC was readily detected in the input samples but not in the immunoprecipitates (Fig. 7B), suggesting that it was not stably associated with p-PLC-γ1(S1248). When coimmunoprecipitation studies were performed with cellular fractions of the mock-infected Golgi apparatus using p-PLC-γ1(S1248)-specific monoclonal antibody (Fig. 7C), gD protein could not be immunoblotted in the immunoprecipitate, further validating the specificity of these IP experiments.

Collectively, these data suggested that p-PLC-γ1(S1248) specifically associates with viral protein gD in the Golgi apparatus.

### p-PLC-γ1(S1248) and virion-associated proteins colocalized to the plasma membrane

**Fig 8:**
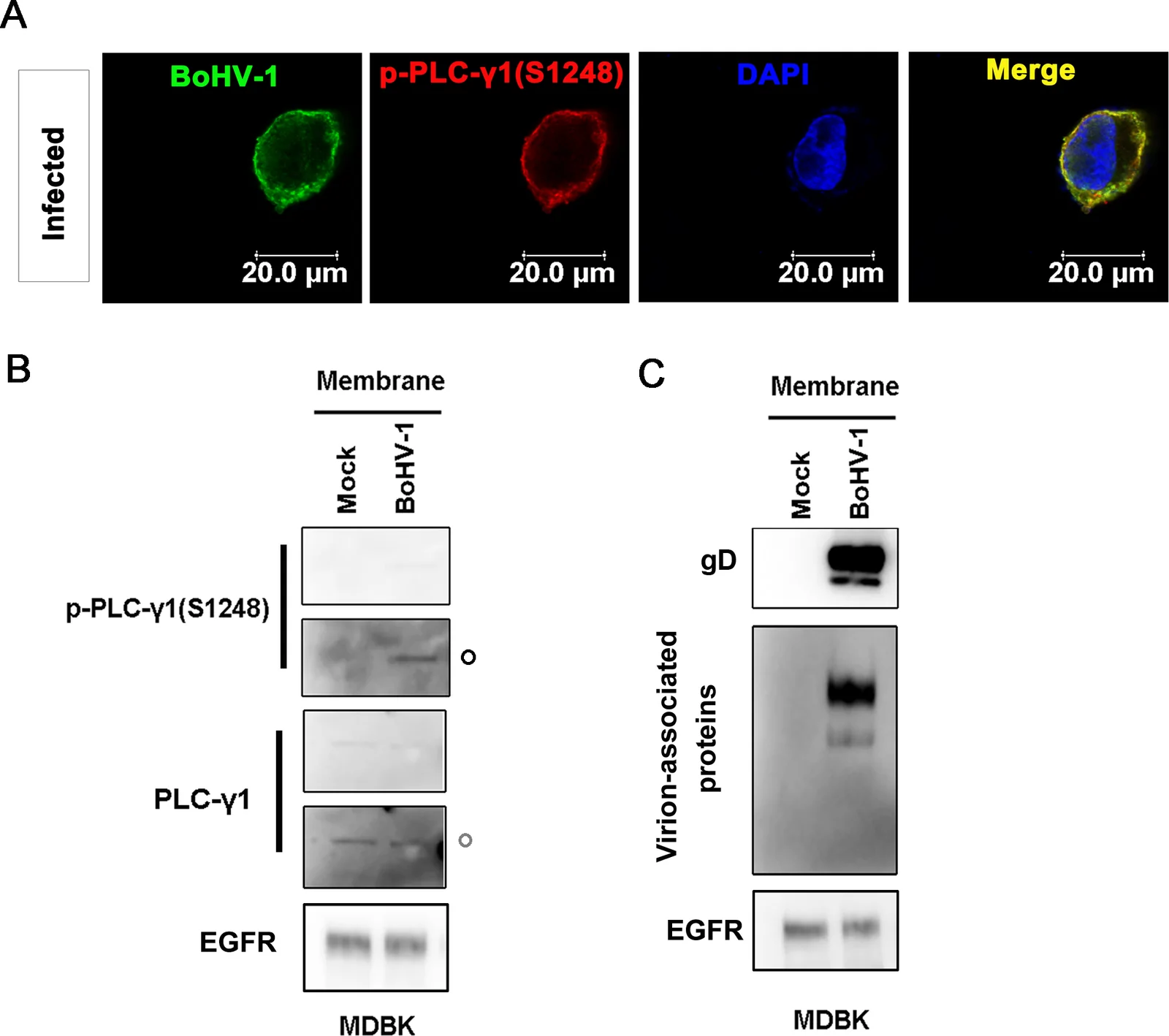
Detection of p-PLC-γ1(S1248) from plasma membranes. (A) Detection of virion-associated proteins and p-PLC-γ1(S1248) in virus-infected MDBK cells with IFA. After infection with BoHV-1 (MOI = 0.1) for 24 hours, MDBK cells were immunostained via using an antibody against virion-associated proteins (green) and an antibody against p-PLC-γ1(S1248) (red), then visualized by confocal microscopy. Nuclei were stained with DAPI (blue). The images were captured using confocal microscopy. (B) Detection of BoHV-1 virion-associated proteins and p-PLC-γ1(S1248) in plasma membranes. MDBK cells in 60 mm dishes were mock infected or infected with BoHV-1 (MOI = 0.1) for 24 hpi. Then plasma membranes were purified using a commercial kit (Beyotime Biotechnology, cat# P0033). Then p-PLC-γ1(S1248), PLC-γ1 (B), as well as the virion-associated proteins, and viral envelope glycoprotein gD (C) were detected by immunoblots. The protein levels of EGFR were probed as the protein loading control. The data shown are representative of three independent experiments.

In addition to localizing to the Golgi apparatus and forming puncta, we noticed that a subset of p-PLC-γ1(S1248) and virion-associated proteins may colocalize to the plasma membranes without showing typical puncta in some MDBK cell populations via a confocal microscope assay (Fig. 8A). To substantiate this observation, the plasma membrane fractions were purified for the detection of both p-PLC-γ1(S1248) and virion-associated proteins using Western blotting analysis. A faint band of p-PLC-γ1(S1248) was recognized by the antibody, which was readily detected in the virus-infected membrane fractions but not in that of uninfected control upon prolonged exposure (Fig. 8B, denoted by the black circle), suggesting that the plasma membrane-associated PLC-γ1 is not activated prior to viral infection. The PLC-γ1 bands were clearly developed in both samples after prolonged exposure, and PLC-γ1 was expressed at lower levels in virus-infected plasma membranes (Fig. 8B, denoted by the gray circle). The host-shutoff effects of BoHV-1 infection at late stages may account for the decreased protein levels of PLC-γ1 in the plasma membrane fractions. These data suggested that the virus infection activated PLC-γ1 in the plasma membrane fractions, as demonstrated by the increased accumulation of p-PLC-γ1(S1248), which was not due to alteration of total PLC-γ1 protein levels. Both viral protein gD and virion-associated proteins were exclusively detected in the fractions of virus-infected plasma membrane from MDBK cells (Fig. 8C), which supports the findings that p-PLC-γ1(S1248) and virion-associated proteins colocalized to the cell membranes via IFA (Fig. 8A). Based on these data, we may speculate that the activated p-PLC-γ1(S1248) associates with virions in the plasma membrane.

### p-PLC-γ1(S1248) associated with the released virions

**Fig 9:**
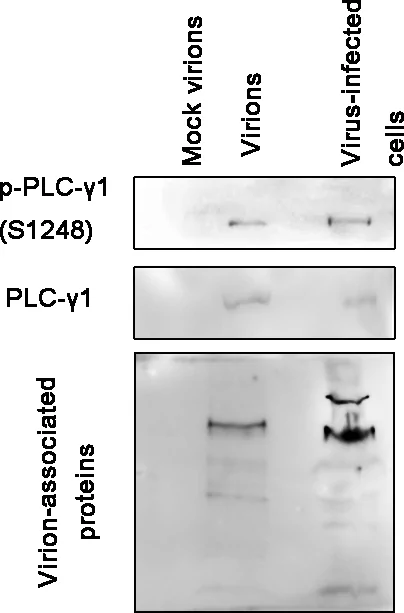
Detection of p-PLC-γ1(S1248) in the released virions. MDBK cells in 100 mm dishes were either mock-infected or infected by BoHV-1 (MOI = 0.1) for 24 hours. The supernatants were collected for the purification of the released virions by ultracentrifugation. Then the products of both virions and mock virions were subjected to Western blot to probe p-PLC-γ1(S1248), PLC-γ1, and virion-associated proteins, respectively. Cell lysates of virus-infected MDBK cells (MOI = 0.1) were used as a positive control. The data shown are representative of three independent experiments.

Considering that a subset of p-PLC-γ1(S1248) may associate with virion-associated proteins in the plasma membranes (Fig. 8B), we wondered whether p-PLC-γ1(S1248) associates with the released virions. The released viral particles propagated in MDBK cells were purified following a protocol as described elsewhere (27) and were subjected to the detection of p-PLC-γ1(S1248) by Western blotting analysis. Strikingly, we found that both p-PLC-γ1(S1248) and PLC-γ1 can be clearly detected in the released viral particles (Fig. 9), indicating that a subset of p-PLC-γ1(S1248) was attached to the released virions.

Taking these data together, we found that the activated p-PLC-γ1(S1248) interacts with virions in the Golgi apparatus, plasma membranes, as well as extracellular spaces.

### PLC-γ1-specific inhibitor, U73122, traps gD in the Golgi apparatus

**Fig 10:**
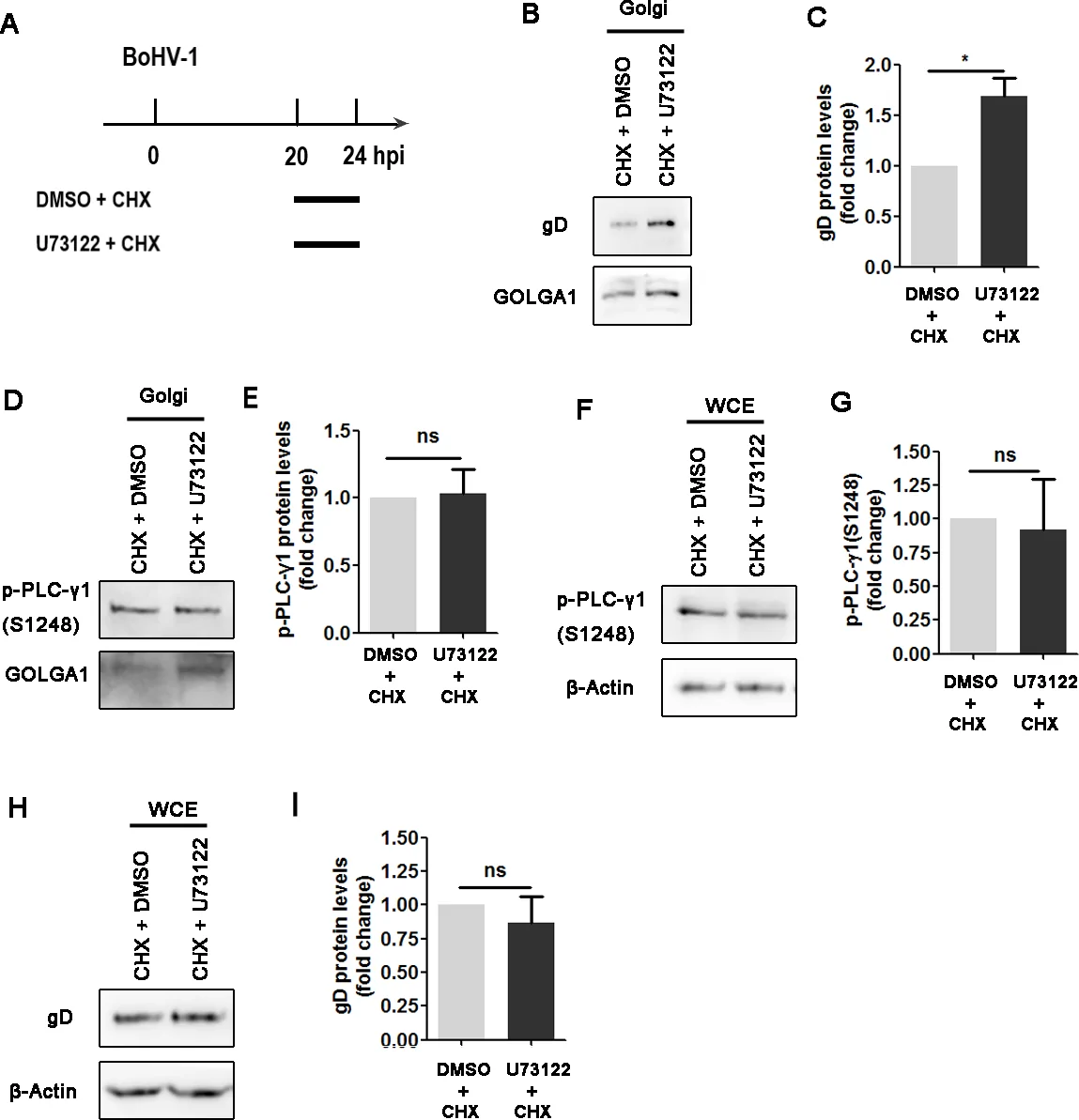
U73122 had effects on the accumulation of gD in the Golgi apparatus. (A) Diagram showing two different treatment manners. MDBK cells in 60 mm dishes were mock infected or infected with BoHV-1 (MOI = 0.1). Virus-infected MDBK cells were treated either with vehicle control dimethyl sulfoxide (DMSO) or U73122 in combination with cycloheximide for a duration of 4 hours prior to the termination of infection. (B–I) After chemical treatment, the cells were collected to purify the Golgi apparatus using commercial purification kit (Beijing Biolabo Technology, cat# HR0247) (B and D) or to prepare the whole cell extracts (WCE) (F and H). Then Western blot was conducted to detect the protein levels of gD and p-PLC-γ1(S1248). The protein gD (B and H) and p-PLC-γ1(S1248) (D and F) were detected in the Golgi apparatus and whole cell extracts, respectively. (C, E, G, and I) The band intensity was analyzed with free software Image J. The data shown are representative results of three independent experiments with error bars indicating standard deviations. Significance was assessed with a Student *t*-test (*P < 0.05, ns: not significant).

Golgi is proposed as the key site of *de novo* HSV-1 capsid envelopment, and egress to the plasma membranes (28). Since viral protein gD is associated with p-PLC-γ1(S1248) in Golgi apparatus (Fig. 7), we wondered whether phospholipase C signaling pathway has effects on the accumulation of gD in this organelle. Here, U73122, a PLC-γ1-specific inhibitor, that works via reduction of agonist-induced Ca^2+^ increases, was employed to treat the virus-infected MDBK cells. Compound treatment was conducted for a duration of 4 hours prior to the termination of the infection (from 20 to 24 hpi) as shown in the diagram of Fig. 10A. During the chemical treatment, cycloheximide (CHX) was included to block the *de novo* synthesis of viral protein gD, then the Golgi apparatus was purified for the subsequent analysis. As a result, gD protein levels were increased in the Golgi apparatus due to the treatment of U73122 (Fig. 10B). Relative to the mock-treated control, gD protein levels were increased to approximately 1.5-fold by U73122 (Fig. 10C), while U73122 has no effects on the accumulation of p-PLC-γ1(S1248) protein levels in the virus-infected Golgi apparatus (Fig. 10D and E). In addition, the treatment by U73122 had no effects on the protein levels of either p-PLC-γ1(S1248) or gD in the virus-infected cells (Fig. 10F through I). Of note, the *de novo* synthesis of viral protein gD was blocked by cycloheximide, and the increased accumulation of gD in the Golgi apparatus was not due to the variation of total gD protein levels. Taking these data together, we suggested that U73122 treatment leads to gD or virions being trapped in the Golgi apparatus. Since the virions trafficking from the Golgi apparatus to the plasma membranes, where it get released out of the plasma membranes, which made it difficult to accurately conclude the effects that U73122 treatment had on the accumulation of virions on the plasma membranes and virus release.

## DISCUSSION

It has been well characterized that PLC-γ1 may locate at distinct cellular fractions, such as in cell-cell junctions (29), cytosol (30), plasma membranes, as well as ruffle membranes (31). The biological functions of PLC-γ1 may be varied with distinct subcellular locations. For instance, the activated PLC-γ1 in the cytoplasm membranes is required to hydrolyze phosphatidylinositol 4,5-bisphosphate (PIP2) to inositol 1,4,5-trisphosphate (IP3) and diacyl glycerol (DAG); thereby, IP3 promotes intracellular calcium mobilization to trigger keratinocyte differentiation (32), while increased accumulation of nuclear PLC-γ1 induced by NaCl is essential to increase the transcriptional/transactivating activity of TonEBP/OREBP, an osmoprotective transcription factor (33), which is important to suppress the osmotic stress. Here, for the first time, we reported that a subset of PLC-γ1 locates at the Golgi apparatus in uninfected-MDBK cells (Fig. 3). As a Golgi apparatus residing component, PLC-γ1 may have effects on the biological functions of this organelle that need to be characterized in the future. It may perform fundamental roles in BoHV-1 productive infection because during BoHV-1 productive infection at later stages, the accumulation of the activated-p-PLC-γ1(S1248) in the Golgi apparatus is enhanced (Fig. 3), where p-PLC-γ1(S1248) interacts with the viral envelope glycoprotein gD (Fig. 1 and 3 to 5). In addition, both p-PLC-γ1(S1248) and viral envelope glycoprotein gD also colocalized in the plasma membranes (Fig. 8). Golgi apparatus is a key organelle of the secretory pathway that receives cargos synthesized in the endoplasmic reticulum, process them, and then sorts them to the correct intracellular compartment. It has been reported that the Golgi apparatus provides a critical site for HSV-1 glycoprotein sorting, secondary envelopment, and trafficking of progeny virions to the cell surface to complete replication cycles (34). Like HSV-1, the Golgi apparatus is an essential site for BoHV-1 packaging into mature virions (26). We investigated whether the phospholipase C signaling has effects on the traffic of virions out of the Golgi apparatus via using PLC-γ1-specific inhibitor U73122. Interestingly, we found that the treatment of virus-infected cells at later stages of virus infection leads to the accumulation of more gD protein in the Golgi apparatus (Fig. 10). Since the *de novo* synthesis of gD protein was blocked by cycloheximide, we suggested that U73122 treatment traps gD or virions in the Golgi apparatus. Here, we noticed that U73122 did not decrease the protein levels of p-PLC-γ1(S1248) (Fig. 10). This is reasonable because U73122 inhibits PLC signaling by blocking intracellular Ca^2+^ mobilization.

Thus, we speculate that the activated PLC signaling pathway may facilitate virus trafficking out of the Golgi apparatus. It has been reported that DAG, one of the PLC-γ1-catalyzed products, traps HSV-1 in Golgi apparatus (22), which supports our findings that U73122 treatment leads to gD of BoHV-1 to be trapped in the organelle.

Like HSV-1, the Golgi apparatus is an essential site for BoHV-1 packaging into mature virions (26). During the preparation of this draft, a paper has published and reported that BoHV-1 glycoprotein M mediates the translocation of VP8 to the Golgi apparatus for packaging of VP8 into virions (35). Here, for the first time, we showed that p-PLC-γ1(S1248) is associated with virion-associated proteins, in the Golgi apparatus, the plasma membranes, and the released virions. These novel findings suggested that PLCγ1 signaling may regulate virus replication in a mechanism involving an association with virion. Whether this association affects virus packaging of gD into virions deserves independent studies in the future.

It has been reported that PLC-γ1 is enriched in plasma membranes to mediate various biological functions, such as activation of T-cell receptor (TCR) signaling transduction (36 – 38). Here, with an independent study using MDBK cells, we confirmed that PLC-γ1 is abundantly expressed in plasma membranes (Fig. 8B). Of note, it has been reported that the presence of PLC-γ1 in membrane structures and its access to the substrate appear to be transient and are followed by rapid incorporation into intracellular vesicles, leading to downregulation of the PLC activity (39). While in this study, we found that the accumulation of p-PLC-γ1(S1248) in the plasma membranes is obviously elevated in response to BoHV-1 productive infection at later stages (Fig. 8B), which colocalized with virion-associated proteins identified by confocal microscope (Fig. 8A). It seems that the activated p-PLC-γ1(S1248) that is associated with virion-associated proteins in plasma membrane fractions is stabilized, which warrants extensive studies in the future.

Strikingly, we found that both PLC-γ1 and p-PLC-γ1(S1248) can be detected in the released purified virions (Fig. 9). Since part of the viral envelope is derived from cellular membranes, it is reasonable that the virus containing p-PLC-γ1(S1248) was obtained via envelopment processes. Although we currently could not reveal the mechanisms of how and why p-PLC-γ1(S1248) is associated with the virions, this is an interesting finding because to our knowledge p-PLC-γ1(S1248) is the first host protein to be consistently associated with BoHV-1 virions during virus productive infection. These data may shed light on the novel roles of PLC-γ1 signaling pathways that played in BoHV-1 productive infection.

In summary, in this study, for the first time, we showed that BoHV-1 productive infection at later stages activates PLC-γ1 signaling, which promotes virions’ trafficking out of the Golgi apparatus, and the activated p-PLC-γ1(S1248) consistently associates with virions, representing a novel mechanism to regulate the virus replication deserving to be determined in the future.

## MATERIALS AND METHODS

### Cells and viruses

MDBK and A549 cells (purchased from Chinese Model Culture Preservation Center, Shanghai, China) were routinely passaged and maintained in Dulbecco's modified Eagle medium (DMEM) supplemented with 10% fetal bovine serum. BoHV-1 stain NJ-16-1 isolated from bovine semen samples (40) was propagated in MDBK cells. Aliquots of virus stocks were stored at −70°C until use.

### Antibodies and reagents

The following antibodies were used in this study: p-PLC-γ1(Ser1248) rabbit mAb (cat# 8713), PLC-γ1 pAb (cat# 2822S), β-actin rabbit mAb (cat# 4970), horseradish peroxidase (HRP)-conjugated goat anti-mouse IgG (cat# 7076), as well as HRP-labeled goat anti-rabbit IgG (cat# 7074) were purchased from Cell Signaling Technology (Danvers, MA, USA). BoHV-1 gD mAb (cat# 1B8-F11) and goat anti-BoHV-1 serum (cat# PAB-IBR) were provided by VMRD Inc. (Pullman, WA, USA). EGFR pAb (cat# A11577) and GOLGA1 rabbit pAb (cat# A14688) were bought from Abclonal Technology (Woburn, MA, USA). GP73/GOLPH2 mouse mAb (cat# 66331-1-lg) was provided by Proteintech (Rosemont, IL, USA). Donkey anti-goat IgG H&L (HRP) (ca# ab97110), Alexa Fluor 647-conjugated goat pAb to rabbit IgG (cat# ab150079), and Alexa Fluor 488-conjugated donkey anti-goat IgG (cat# ab150129) were provided by Abcam (Cambridge, UK). Alexa Fluor 488-conjugated goat anti-rabbit IgG (H + L) (cat# A-11008) and Alexa Fluor 633-conjugated goat anti-mouse IgG (H + L) (Invitrogen, cat# A-21052) were provided by Invitrogen Life Technologies (Waltham, MA, USA).

### Western blotting analysis

Cell lysates of either whole cell extracts (WCE) or cellular fractions, including plasma membranes and Golgi apparatus were prepared using radioimmunoprecipitation assay (RIPA) lysis buffer (1× phosphate buffered saline [PBS], 1% NP-40, 0.5% sodium deoxycholate, 0.1% SDS) supplemented with protease inhibitor cocktail. They were boiled in Laemmli sample buffer for 5 minutes, subsequently subjected to separation on SDS-PAGE (8% or 10%), and then transferred to polyvinylidene fluoride membranes. Immuno-reactive bands were developed using Clarity Western ECL Substrate (Bio-Rad, cat# 1705061).

For the designated studies, the band intensity was quantitatively analyzed with free Image J program (https://imagej.nih.gov/ij/download.html) (accessed on 1 December 2020), which was initially normalized to either β-actin or GOLGA1, and the fold change after treatment was calculated. Protein levels in mock-treated cells were arbitrarily set to 1. Significance was assessed with a Student *t*-test by using GraphPad Prism software (v5.0). *P* values of less than 0.05 (*P < 0.05) were considered significant for all the calculations.

### Immunofluorescence assay

MDBK cells in 8-well chamber slides (Nunc Inc., Naperville, IL, USA) were mock infected or infected with BoHV-1 (MOI = 0.1). After infection for 24 hours, cells were fixed with 4% paraformaldehyde in PBS for 10 min at room temperature, permeabilized with 0.25% Triton X-100 in PBS for 10 min at room temperature, and blocked with 1% bovine serum albumin (BSA) in PBS with Tween-20 (PBST) for 1 hour followed by incubation with the indicated antibodies in 1% BSA in PBST overnight at 4°C. After three washings, cells were incubated with secondary antibody labeled with distinct fluorescent dyes for 1 hour in the dark at room temperature. After three washings, DAPI (4′,6-diamidino-2-phenylindole) staining was performed to visualize nuclei. Slides were covered with coverslips by using an antifade mounting medium (Electron Microscopy Sciences, cat# 50-247-04). Images were captured using a confocal microscope (Leica).

### Immunoprecipitation assay

Cellular fractions of Golgi apparatus derived either from virus-infected cells or mock-infected cells were clarified by centrifugation at 16,000 rpm for 10 min and incubated with Dynabeads protein A beads (Life Technologies, cat# 10001D), which have been precoated with either p-PLC-γ1(Ser1248) mAb or isotype IgG by incubation for 1 hour at room temperature with rotation. After overnight incubation at 4°C with rotation, Dynabeads were collected using a magnet (DynaMag) (Life Technologies, cat# 12321D). After three washings with PBS, beads were boiled in SDS-loading buffer and subjected to Western blot.

### The treatment of virus-infected cells with U73122 and and CHX

MDBK cells of confluent in 100 mm dishes were infected with BoHV-1 (MOI = 0.1) for 20 hours. Then the virus-infected MDBK cells were treated either with vehicle control DMSO or U73122 (5 µM) along with cycloheximide (10 µM) for 4 hours. After treatment, the cells were collected to prepare the WCE by using RIPA lysis buffer or to purify the Golgi organelles using a commercial purification kit (Beijing Biolabo Technology, cat# HR0247).
